# Correction: Keeping the “R” in LARC (long-acting reversible contraception): Measuring client-centered implant removal services in sub-Saharan Africa

**DOI:** 10.1371/journal.pgph.0006863

**Published:** 2026-07-13

**Authors:** Celia Karp, Katherine Tumlinson, Brooke W. Bullington, Linnea A. Zimmerman, Leigh Senderowicz

The following information is missing from the Funding statement: This study was supported by NIH grant number (K01HD113818) from the NICHD K01.
